# Patient-Specific Orthognathic Solutions: Expert Opinion on Guidelines and Workflow

**DOI:** 10.3390/cmtr18010012

**Published:** 2025-02-06

**Authors:** Alf L. Nastri, Isaac Liau, Jaewon Heo, Alexander Schramm

**Affiliations:** 1 Department of Oral & Maxillofacial Surgery, Royal Melbourne Hospital, 300 Grattan St., Parkville, VIC 3050, Australia; isaac.liau@gmail.com (I.L.); jaewon727@gmail.com (J.H.); 2 Klinik für Mund-, Kiefer- und Gesichtschirurgie, Albert-Einstein-Allee 11, 89081 Ulm, Germany; alexander.schramm@uni-ulm.de

**Keywords:** orthognathic surgery, bimaxillary osteotomy, virtual surgical planning, patient-specific implants, workflow, guideline

## Abstract

This document outlines guidelines for the use of three-dimensional virtual surgical planning in orthognathic surgery, with relevance to data acquisition, clinical diagnosis, data workflow sequencing, and operative considerations. A detailed description regarding fundamental principles of orthognathic assessment and planning is beyond the scope of this paper.

## 1. Introduction

Treatment planning for orthognathic surgery is complex and has traditionally relied on the surgeon’s experience, plain film radiography, and plaster dental casts mounted on semi-adjustable anatomical articulators to perform model surgery. However, these methods do not provide a comprehensive representation of the three-dimensional skeletal anatomy of the facial skeleton, thereby making it difficult to accurately visualize complex surgical movements with occlusal splint-based transfer only.

Model surgery is a critical component in planning orthognathic surgery. In complex bimaxillary surgery, traditional model surgery requires multiple laboratory-based steps that are both time-consuming and potential sources of error. The clinician’s ability to assess the patient, obtain accurate records, and execute model surgery with planned movements is crucial for predictable outcomes.

In the past decade, computer-assisted virtual surgical planning (VSP) for the treatment of orthognathic deformities has advanced significantly. The VSP process addresses many of the limitations of traditional approaches mentioned above [1]. It also allows the transfer of 3D virtual treatment planning to the operating theatre, by using computer-aided design (CAD)/computer-aided manufacturing (CAM)-generated occlusal wafers and positioning systems, navigation, customized surgical guides, and patient-specific implants (PSI).

Digital treatment planning and patient-specific cutting guides/implants enhances the planning and execution of complex orthognathic procedures. Other advantages of VSP are reduced preoperative planning/preparation time, 3D visualization of surgical movements, and identification of anatomical variations that may impact the procedure (for example, tooth roots, nerve position, cortico-cancellous anatomy, or bony interferences). It also serves as a visual aid for patients, improving information delivery, including the use of postoperative simulations. Surgical time may also be reduced, especially with patient-specific cutting guides and implants [1,2,3].

While 3D virtual planning is widely used in orthognathic surgery, it supplements, rather than replaces, thorough clinical assessment and individualized management. While VSP workflows may save time and improve accuracy, associated costs must be considered. Experience in patient evaluation, planning, and surgical execution is essential for success, regardless of the planning method used.

## 2. Workflow

**Figure 1:**
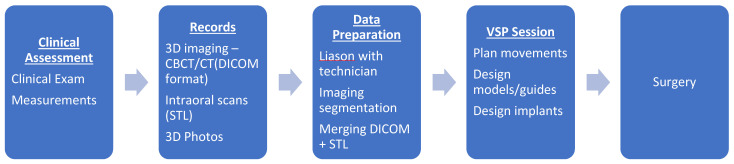
VSP workflow chart. CBCT, cone-beam computed tomography; CT, computed tomography; DICOM, Digital Imaging and Communications in Medicine; STL, stereolithography; VSP, virtual surgical planning.

The VSP workflow is similar to the traditional model surgery pathway and outlined in Figure 1. A clinical examination combined with baseline radiographic assessment (including orthopantomogram [OPG] and lateral cephalogram) determines the surgical plan. Additional records are required to perform VSP, but it replaces the need to perform manual model surgery and fabrication of occlusal wafers. It also provides additional adjuncts otherwise not available in traditional methods.

The additional record requirements for VSP include either fine-slice computed tomography (CT), or high-resolution cone-beam computed tomography (CBCT). Impressions can be replaced with intraoral scans or scanned dental models to improve occlusion and wafer accuracy. Standard clinical photographs are helpful, but 3D photographs for soft tissue overlay are also available to improve the 3D visualization.

The next data preparation phase involves gathering digital data and uploading it to VSP software for processing, alignment, and fusion. Images are processed for structural segmentation and surface rendering to aid virtual surgery. This phase is usually performed by clinical engineers who are familiar with the process and software programme. Before starting virtual surgery, surgeons and engineers must ensure the condyle is seated, the head is in the patient’s natural position, and the facial midline is aligned as per clinical assessment.

After the virtual surgical movement has been performed and verified, the next step is to design the surgical splints (final and intermediate wafers) and adjuncts (e.g., palatal splint for segmental cases). If a PSI is involved, the design of the cutting guide and implant must be discussed. Once the VSP report is reviewed, the associated products can be approved for 3D printing, ensuring accurate transfer of VSP information to the surgery.

## 3. Clinical Assessment

This section does not cover the fundamental principles of orthognathic assessment. For a comprehensive understanding of these principles and treatment planning, refer to “Essentials of Orthognathic Surgery” by Reyneke or “Dentofacial Deformities: Integrated Orthodontic and Surgical Correction” by Epker, as the core principles remain unchanged regardless of the planning method.

All the key parameters involved in orthognathic assessment should be recorded so that they can be documented and used in treatment planning. In a complex case with difficult asymmetry, cant, midline discrepancy, significant anatomical variations in both soft and hard tissue, it can be difficult to document and translate into conventional model surgery.

The key point to understand is that the 3D information gathered during the VSP should supplement the surgeon’s clinical assessment, rather than override the observations and measurements obtained during the physical assessment. For instance, the midline reference point should be established based on the clinical assessment of the patient’s overall profile, rather than solely relying on the skeletal midline observed in the VSP.

The assessment of occlusion is crucial, regardless of the planning method, particularly when planning for maxilla-first bimaxillary orthognathic surgery. Patients with significant Class II malocclusion may exhibit posturing, which can adversely affect centric relation (CR) if not properly controlled and monitored. It is also essential to assess any shift in centric occlusion (CO) from CR (CR/CO shift). This phenomenon is more commonly observed in Class III patients with occlusal interference. If not managed effectively, patients may easily slide whilst attempting to take bite registration. The VSP method provides an additional safety measure by allowing for the verification of condylar position during occlusion. Therefore, if the CR record is found to be incorrect, it can be re-taken, or the condyle can be virtually seated into the glenoid fossa by VSP engineers.

Another important aspect to consider is soft tissue assessment and how the patient’s soft tissue profile will change based on the planned surgical movements. VSP with soft tissue overlay can predict a certain degree of soft tissue change according to an algorithm established in various studies. A study by Awad et al. demonstrated that soft tissue predictions can be as accurate as 69–96%, depending on the facial subunit, with greater accuracy observed in the upper facial subunits compared to the lower ones [4]. Numerous variables, such as age, ethnicity, soft tissue thickness, and the degree of movement, can significantly impact the final soft tissue position. Particularly, when planning to use cutting guides and PSIs, additional attention should be given to predicting the likely soft tissue changes, as it is difficult to alter from the planned movements intraoperatively.

## 4. Data Acquisition

Following clinical assessment and the formulation of a treatment plan, the traditional model surgery pathway requires clinical photographs of extraoral and intraoral images, impressions to produce study models, as well as facebow and CR occlusal records to accurately mount the models on the articulator.

For the VSP pathway, a CBCT or CT scan is required to facilitate virtual surgery. It is essential to adhere to a scanning protocol to maximize the accuracy of planning, models, guides, and implants. Additionally, it is recommended that the scan be no older than four months at the time of design, and surgery should not be performed later than six months after the scan.

**Figure 2:**
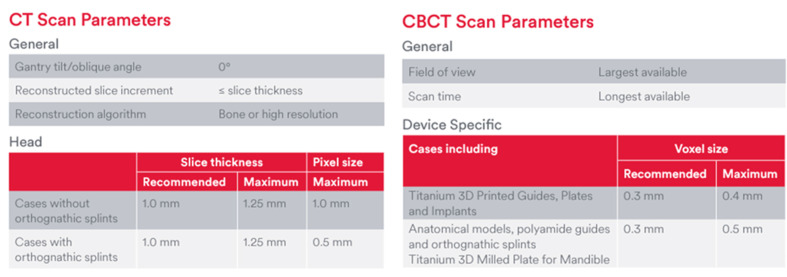
CT/CBCT scan protocol (as per the DepuySynthes/Materialize scan protocol). CBCT, coned-beam computed tomography; CT, computed tomography.

The recommended protocol for the CT scan includes a slice thickness of 1 mm and a maximum pixel size of 1 mm. The patient should be positioned with the gantry tilted/oblique angle of 0° and the occlusal plane aligned parallel to the gantry. A field of view should include the entire facial skeleton, including orbits and temporomandibular joints (TMJs). A bone or high-resolution reconstruction algorithm should be utilized. The axial reconstruction should have a slice thickness of 1 mm or less, with a matrix size of either 512 × 512 or 768 × 768. For the CBCT scan, the recommended protocol specifies a voxel size of 0.3 mm, utilizing the longest scan time available and the largest possible field of view to maximize accuracy (as per the protocol show in Figure 2; please confirm the recommendations of the local VSP software requirements). It is also essential to ensure that the scan file is in uncompressed DICOM (Digital Imaging and Communications in Medicine) format, with no post-processing or reformatting applied.

**Figure 3:**
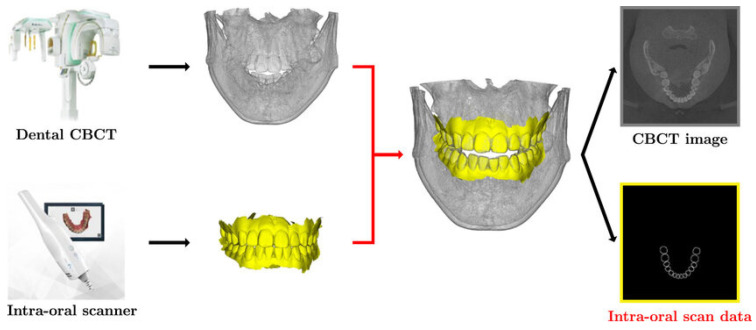
Image showing how to merge the intraoral scan with CBCT (Image from Hyun et al., 2022 [5]). CBCT, coned-beam computed tomography.

To enhance the accuracy of treatment planning, occlusal imaging can be uploaded. The most precise method is to use an intraoral scan merged with a CT/CBCT scan (Figure 3). If an intraoral scanner is unavailable, an impression can be taken, and the study model can be scanned using an optical scanner. It is also essential to learn how to assess and obtain a centric relation bite registration. This can be accomplished with an intraoral scanner, or a standard bite registration can be sent along with the impression for the study models to be scanned in occlusion. Additionally, while it is beneficial to assess and establish a final occlusion record to be sent for the VSP, it is not mandatory; experienced engineers and surgeons can establish the final occlusion during the VSP. These occlusal images are submitted in STL (stereolithography) format.

## 5. Data Preparation

Following the completion of the data acquisition phase, the records data are now ready to be prepared for VSP. Note that the clinical assessment and formulation of a surgical plan must be complete prior to proceeding with VSP.

CT imaging data are obtained in uncompressed DICOM format for exportation of the dataset into VSP software applications. The dataset must first be verified to check that the scan is of sufficient quality to proceed with accurate planning as per the previously highlighted parameters with no movement artefact.

Dental occlusal records digitized in STL format are uploaded into the virtual workspace. Dental study models are useful in the clinical assessment phase to identify additional occlusal parameters (e.g., arch coordination, transverse discrepancies, or premature occlusal contacts in the final occlusion), and are a recommended adjunct diagnostic tool regardless of whether the occlusion is set digitally or manually. Therefore, if dental records are digitally obtained via intra-oral scanning, they can be printed as a study model to allow tactile review of the occlusion by the surgeon.

The virtual planning dataset is completed by merging the STL dental occlusal records with the CT scan (using CT registration of the dentition as the site for overlay). Note that, in cases where the final occlusion is set and supplied by the surgeon for planning, any sites of occlusal interference reduction performed before VSP should be recorded as these will not be demonstrated in the final planning report. Following overlay of the dentition on the CT, digitized jaw relation records are then used to confirm accurate seating of the condyle in centric relation, which can be virtually corrected as required.

Optionally, 3D photographic records (if available) can also be merged onto the virtual dataset in a similar manner for use in soft tissue prediction modelling.

The complete dataset then undergoes segmentation. This process involves the placement of virtual osteotomy planes at equivalent sites in the facial skeleton to those performed during in vivo surgery. Following segmentation, individual sections of the facial skeleton can then be moved during virtual planning to replicate orthognathic surgical movements. Prior to the planning session, the following information must be communicated to the engineer performing the segmentation:

- Type of operation (single jaw vs. bimaxillary);
- Additional sites of segmentation (e.g., segmental maxillary surgery);
- Alternative osteotomy design (e.g., vertical ramus osteotomy, inverted-L ramus osteotomy, higher level LeFort maxilla, or zygomatic osteotomies).

**Figure 4:**
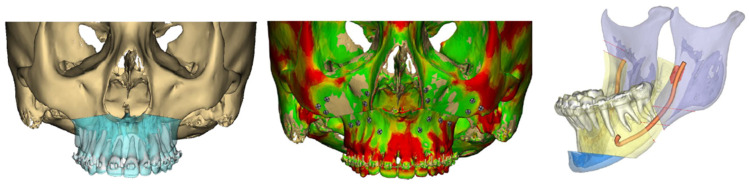
Example of segmentation process, bone thickness assessment, and nerve tracing.

During the segmentation process, key anatomical structures such as the inferior alveolar nerve canal can also be identified and mapped three-dimensionally with relevance to osteotomy sites. For the purposes of fixation screw placement, bone thickness can also be measured and represented via a heat-map reconstruction (Figure 4).

**Figure 5:**
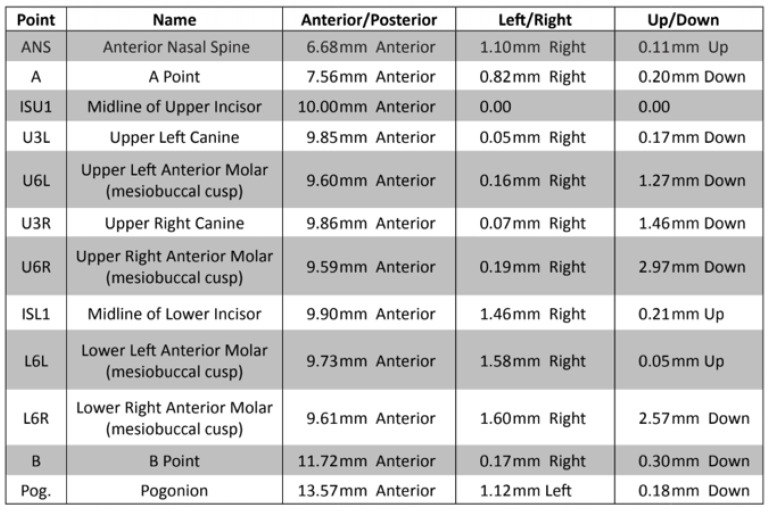
Example of key lateral cephalogram landmarks and associated movements.

Finally, 3D analytic landmarks (see Figure 5) are also marked and identified, to provide movement analysis reports at the conclusion of the planning session.

## 6. Virtual Surgical Planning Session

**Figure 6:**
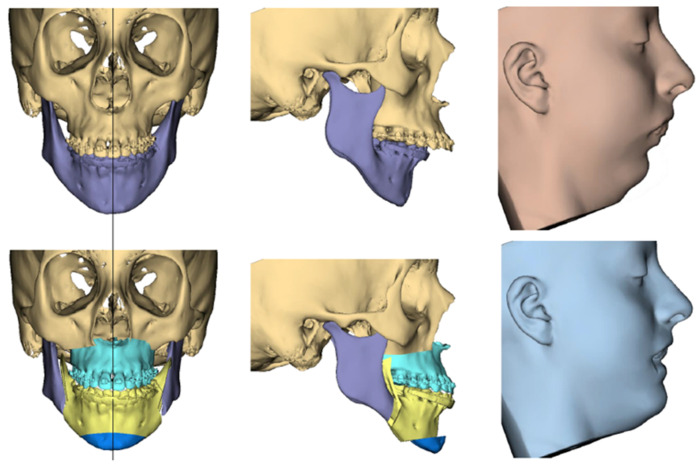
Top images showing original patient position, and bottom images showing the changes following completion of 3D simulation of surgical movements including the soft tissue prediction.

**Figure 7:**
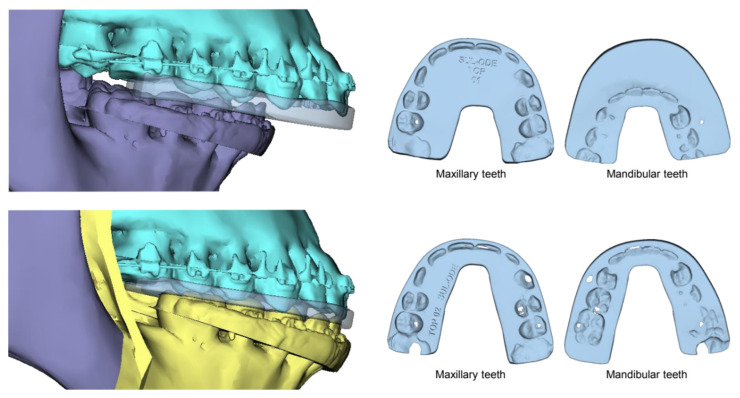
An example of intermediate (top) and final splint (bottom) used for bimaxillary osteotomy (maxillary first surgery case).

VSP typically proceeds in the following sequence (with variations according to surgeon preference) (see Figure 6 and Figure 7):

1. Confirm 3D natural head position.
2. Confirm condylar position in centric relation.
3. Define dentofacial deformity (crosscheck with clinical assessment measurements):
  - Facial midline.
  - Dental midlines.
  - Cant deformities.
4. Establish final occlusion either by
  - Scanning pre-articulated occlusion sent by the surgeon, or
  - Virtual setting of occlusion.
5. Check and record occlusal interference depth.
6. Maxillary movement:
  - Frontal view—Midline and cant.
  - Inferior view—Yaw.
  - Sagittal axis—Anteroposterior, occlusal plane, vertical change.

*(Intermediate position now set).*

7. Mandibular movement:
  - Mandibular distal segment is merged with the final occlusion position.
  - Proximal segment adjustments:
    - Alignment of lower border (condylar rotation in sagittal plane).
    - Alignment of buccal cortex (condylar rotation in axial plane).
  - Identification of bony interferences—adjust proximal segment position if suitable.

*(Final occlusion position now set).*

8. Genioplasty movement (if required):
  - Frontal (midline, cant, vertical).
  - Yaw.
  - Anteroposterior.

## 7. Surgical Guide Production

Following the completion of the virtual surgical movements, the plan is then ready to be translated to the patient via production of surgical guides. These can be classified into the following protocols:

1. Splint-only transfer.
2. PSI-only transfer.
3. Combined PSI and splint-based transfer (majority of bimaxillary PSI cases).

### 7.1. Splint Only Transfer

Occlusal splints are constructed in a manner similar to traditional model surgery. The position of the intermediate and final jaw positions is used to guide the design of 3D printed plastic splints. The following points around splint design should be communicated to the engineer as part of the VSP session, largely based on surgeon preference:

- Maxilla vs. mandible first surgery protocol.
- Extent of arch coverage (full arch vs. premolar-premolar).
- Flange thickness (+/− incorporation of wiring holes).
- Segmental maxillary surgery:
  ○ Separate palatal splint vs. incorporation into intermediate/final splints.
  ○ Soft tissue relief to minimize pressure on palatal vascular pedicle.

### 7.2. Patient-Specific Implant Transfer

PSIs utilize the principle of using a customized cutting guide in combination with a customized osteosynthesis plate implant to position the bone segment in the planned position. The virtually planned osteotomy planes, with corresponding areas of bone reduction (if required from the VSP movements) are translated to the patient through the cutting guide. Predictive fixation holes are used in combination with the three-dimensional shape of the custom plate to position the segment accurately in the planned position.

During the VSP session, the design process is “reverse engineered” from the final surgical position by designing the PSI first. The following steps to design the PSI are performed:

1. Decision on single-piece vs. multi-piece PSI.
2. Placement of PSI footplate on sound bone surfaces:
  - Decision on thickness of plate and position of interconnecting bars.
3. Identification of fixation screw hole positions, determined by
  - Bone thickness available.
  - Strength of fixation required.
  - Surgical access.

**Figure 8:**
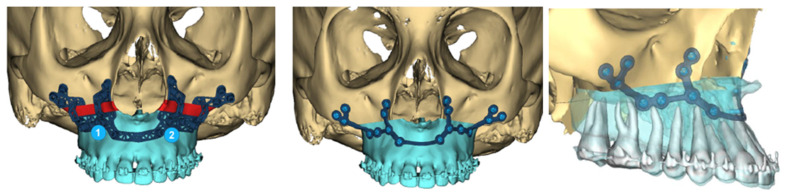
Images showing the titanium cutting guide with predictive holes, which transfers to a patient-specific titanium implant. The positions of the screws are designed to avoid the roots as well as the thin bones highlighted on the heat map seen on Figure 4 (right).

Once the final PSI has been designed, the surgical cutting guide is then designed to place the osteotomy and fixation hole positions in the correct position, derived by the VSP software package. Cutting guides can be constructed out of either plastic (nylon) or titanium (Figure 8). Nylon guides are less costly to produce; however, they are of larger size and retain some inherent flexibility, which can reduce the accuracy of predictive osteotomy positions. Regardless of the guide material, the following steps are performed in cutting guide design during the VSP session:

1. Inclusion of cutting plane at planned osteotomy site.
2. Incorporate areas of bone removal (if required).
3. Ensure sufficient guide extension over 3D landmarks for accurate positive seating—include anti-rotational features:
  - Mandibular cutting guides can be difficult to position accurately due to a lack of defining surface landmarks; this problem can be addressed through the design of a tooth-borne cutting guide.
4. Positioning of cutting guide fixation holes (at least two per side):
  - Ideally should be separate to predictive hole positions, unless insufficient bone volume for separate hole sites.
5. Positioning of predictive screw hole positions:
  - Placed in sufficiently thick bone to accommodate 4–6mm length screws.
  - Position sufficiently distant from osteotomy line.
  - Avoid placement near key anatomical structures (nerves or tooth roots).
  - Consider limitations of surgical access for both screw position and angulation.

For cases where a PSI is required to perform genioplasty movements, the same process is repeated for this bone segment.

The use of PSI over splint-only orthognathic surgery provides several advantages and disadvantages (Table 1).

## 8. Virtual Surgical Planning Report

In the workflow where external companies perform the VSP and PSI/guide construction, an approval process is applied, whereby the treating surgeon approves the virtual plan prior to production. The VSP is communicated to the surgeon in the form of an electronic report document, which provides the following useful clinical information to the surgeon:

- Movement analyses.
- Bone contact points:
  ○ Interference depth requiring bone removal during osteotomy.
  ○ Downgraft positions and predicted graft size.
- Occlusal interference depth.
- Bone thickness map.
- Nerve position measurements.
- Tooth root position measurements.
- Soft tissue prediction modelling.
- Airway prediction modelling.

## 9. Intraoperative Considerations for Virtual Surgical Planning for Orthognathic Surgery

Following the completion of VSP, the planned movements are then transferred in vivo to the patient in the operating theatre. Standard principles apply with regard to anesthesia, patient preparation, and positioning. The operative sites are exposed in the typical fashion with transoral mucosal incisions and subperiosteal degloving of the maxilla and mandible. In splint-only VSP cases, the orthognathic procedure is subsequently completed in the usual manner with “freehand” placement of osteotomy cuts, and fixation of bone segments with stock plates using the custom printed splints as surgical guides for accurate placement of the occlusion in intermaxillary fixation. Utilization of the three-dimensional movement analysis obtained during VSP allows the surgeon to perform judicious bony interference removal where required, and to confirm the correct placement of the osteotomized segments.

In orthognathic cases utilizing a PSI, this standard operative protocol needs to be modified to accommodate the PSI workflow. The typical PSI operative workflow for a double jaw case involves the following:

1. General anesthesia and patient preparation.
2. Surgical exposure—transoral incision with mucoperiosteal flap elevation of maxilla and mandible.
3. Application of maxillary cutting guide and temporary guide fixation with screws.
4. Perform osteotomy cuts with saw/bur/piezoelectric device along cutting guide planes (+/− bony interference removal).
5. Drill predictive holes as per cutting guide.
6. Remove cutting guide.
7. Complete osteotomy cuts in area of cutting guide connecting bars.
8. Lefort 1 osteotomy mobilization in standard manner.
9. Maxilla placed into intermediate splint and intermaxillary fixation (IMF; optional).
10. PSI inserted and passive seating confirmed.
11. Secure PSI with screws in predictive fixation hole positions.
12. Release IMF.
13. Proceed with mandibular osteotomy cuts as per standard protocol

(If mandibular PSI is also used, repeat steps 3–12 in mandible).

14. Genioplasty if indicated.

**Key considerations in the PSI surgical workflow are:**

- Subperiosteal exposure of the operative site must be sufficiently extensive to permit correct seating of both cutting guide and PSI on the bone surface. Common areas that are insufficiently exposed in this process are the superior maxilla, lateral zygomatic body, and degloving inferior to the incision line onto the maxillary alveolus.
- Single-piece cutting guides/PSI typically require a larger mucosal access incision to provide an appropriate path of insertion, and therefore are less compatible with “minimally invasive” incision designs.
- The cutting guide must be intimately applied to the underlying bone surface and seated passively during guide fixation screw placement. Common sources of error in placement are:
  ○ Insufficient exposure with soft tissue remaining between the cutting guide and bone
  ○ Lack of accurate seating due to inadequate guide adaptation to 3D anatomical landmarks (most commonly encountered with bone-borne mandibular cutting guides).
- Predictive screw hole positions must be protected during the osteotomy and mobilization process, as accidental fracture through predictive holes will render the involved screw hole unsuitable for screw placement. High risk cases can be identified preoperatively during the VSP process by analysis of bone thickness and accommodated for with either placement of the screw positions more distant to the osteotomy plane, or with additional contingency fixation holes placed in the PSI.
- Use of wired IMF and occlusal splints is optional. However, some complex movements (e.g., multi-piece segmental maxillary osteotomies) can be difficult to seat passively with only PSI guidance and an occlusal splint as a secondary positioning guide can be useful. Additionally, in the event of predictive hole loss through accidental fracture, occlusal splints and stock fixation hardware provide a backup option to allow completion of the procedure.
- Intraoperative changes to the surgical plan are not compatible with the use of a PSI.

## 10. Discussion

Orthognathic surgery has been evolving over many decades to become more controlled, accurate, predictable, safer, and faster. Computer-aided VSP has introduced a number of advantages over the traditional approach, such as the ability to assess a patient in 3D with improved accuracy and reduction in preoperative planning time [2,3]. It has also opened up the opportunity to move away from record taking, such as impressions and facebow transfer, which can add another layer of inaccuracy [6,7]. A major advantage of VSP-based planning is that it allows for the incorporation of 3D imaging techniques to completely visualize the preoperative and postoperative skeletal positions in multiple planes. In contrast, model surgery-based protocols rely on accurate clinical assessment of the skeletal deformity based on extrapolations of dental or soft tissue landmarks, which are used as analogues of the underlying skeletal anatomy. There is significant evidence to suggest that VSP-based protocols provide a higher level of accuracy in the final surgical result in all dimensions; however, this is particularly the case in the management of asymmetry deformities (midline alignment rates of 93% vs. 58.5% in favour of VSP), which are harder to assess clinically due to the combined effect of soft and hard tissue anatomy [8]. The use of PSI can further improve the accuracy and surgical time compared to using only splints with stock plates [9,10].

The effect of VSP in reducing time required to complete the planning phase of orthognathic surgery has been studied previously by multiple authors. Overall, there is a statistically significant reduction in the total planning time with a VSP protocol as opposed to conventional model surgery between 31 and 91% [4]. The most impactful time reduction is the reduction or elimination of laboratory-based steps such as facebow mounting, model surgery, and splint fabrication. However, the computer planning time for 3D VSP has been reported as double the time required for 2D conventional analysis and planning by some authors, as there are more landmarks and points of analysis to be considered in VSP [5]. The time required for VSP is also impacted by a learning curve to the adoption of VSP software packages, depending on whether the planning is performed in house by the surgeon, or externally with a third-party company [3,8,11].

PSI-based orthognathic surgery requires highly accurate clinical assessment and appropriate treatment planning decisions, as the high accuracy of PSIs restricts the placement of the maxilla to only the originally planned position. This is mostly advantageous as it reduces the likelihood of operator-dependent error, but it eliminates the ability to introduce intraoperative changes to the surgical plan. Typical cases where this may be required are when the unpredictability of soft tissue changes to incisal display require additional vertical change, or when intraoperative conditions do not permit the originally planned surgical movement such as in cleft cases [12]. Splint-based surgery also allows “contingency planning” where multiple intermediate splint positions can be created; for example, in cleft cases where large maxillary advancements may be difficult, a second intermediate splint with a more conservative movement can be constructed if the initially planned movement is not possible.

Intraoperative changes to the vertical dimension are not possible with the use of single-piece PSIs due to their inherent three-dimensional rigidity of sintered titanium alloy, and the removal of the effect of rotational positioning the maxillomandibular complex as would be performed with splint-based surgery [13,14]. This is advantageous in cases requiring higher control over vertical downgraft movements, or counterclockwise rotations of the occlusal plane. However, if the degree of incisal display requires intraoperative adjustment, this is also not possible with PSI fixation. This can be accommodated for in the VSP stage by the utilization of multiple smaller custom plates (either two or four plate designs), where some degree of flexibility in vertical positioning can be obtained, at the cost of accuracy in positioning.

PSI-based surgery is also particularly useful in cases where condylar positioning is not reproducibly reliable for the purposes of positioning the maxilla–mandibular complex in wired IMF [15]. Situations where this may arise are in large class II cases with a large CO/CR shift, degenerative/post-traumatic changes to the TMJ, or congenital deformity of the ramus–condyle unit (e.g., craniofacial microsomia) [16]. In cases of TMJ arthroplasty/replacement with concurrent orthognathic surgery, use of a maxillary PSI also removes the possibility of introducing incorrect maxillary positioning due to TMJ position [17].

The use of mandibular PSIs in orthognathic surgery should be undertaken with caution, as the lack of defining 3D anatomical landmarks on the buccal cortex of the mandible creates challenges with accurate positioning of both cutting guides and PSIs. This issue can be circumvented with the use of tooth-borne rather than bone-borne cutting guides, although the size limitations of a combined tooth- and bone-borne cutting guide within conventional transoral access incisions will result in incomplete coverage of all the planned mandibular osteotomy sites, and hence a portion of the cut will still need to be performed “freehand” [18]. Additionally, the variability in seating of the condyle reduces the accuracy of positioning the mandibular segments in the planned position. For this reason, the use of PSI only without splints is not recommended in mandible-first surgery protocols [19]. The use of occlusal splints and stock plates in one jaw of a double-jaw operation also allows for adaptation to unforeseen changes in condylar position, or minor inaccuracies in the intermediate position.

Cost considerations also need to be considered in the adoption of VSP technology for orthognathic surgery. In general, the cost of VSP workflow surgery is significantly higher, reported by some authors as nearly double that of traditional techniques [20]. These costs are variable depending on which steps are performed in-house by the surgeon, or outsourced to external commercial third parties (e.g., computer planning, fabrication of surgical splints or PSI) and region-dependent. Common areas of increased cost that are introduced by the VSP technique are in the initial capital investment for acquisition of 3D imaging modalities (CBCT scanner, intraoral digital scanner), software licences for VSP computer programmes, and 3D-printing machines (either for plastic splints or custom titanium). Notably, as this cost increase is largely in relation to capital investment at a single point in time, with long-term use of VSP protocols, the overall reduction in planning time (and therefore surgeon productivity) has been shown to mitigate the initial investment cost [21]. In the author’s local country, local epidemiological data have demonstrated a recent ten-fold increase in health insurer expenditure over a 5-year period, from $1.9 million to $17.2 million, attributed to the adoption of VSP surgical guides and biomodels [22]. The cost to the healthcare system of VSP orthognathic surgery is also impacted by sources of funding, where in the absence of adequate health insurance coverage or public hospital institutional funding, the out-of-pocket costs to the patient for PSI-based surgery are prohibitively expensive.

## 11. Conclusions

Since the establishment of the modern era of orthognathic surgery by Obwegeser in the 1950s, there have been many changes due to ongoing innovations in the field of orthognathic surgery. There is no doubt that VSP and PSI play a positive role in the current paradigm shift away from traditional approaches; however, it is important to recognize that the core principles of orthognathic surgery have not changed. The surgeon’s ability to comprehensively assess the patient and formulate an appropriate orthognathic treatment plan is crucial for achieving successful outcomes. Additionally, surgeons must be aware of both the financial and technical limitations of VSP technology. Consequently, appropriate case selection for the use of patient-specific implants in orthognathic surgery should be carefully considered.

## Figures and Tables

**Table 1:** Advantages and disadvantages of patient-specific implants and splint-only techniques.

	Advantages	Disadvantages
PSI	Increased accuracy Position independent of condylar seating Reduced operative time Increased fixation rigidity Suitable cases ○ Complex movements ▪ Cant, yaw, asymmetry ▪ Segmental maxilla ○ Downgraft ○ Counter-clockwise rotation ○ Unreliable condyle position ○ Large maxillary movement (bulky intermediate splint)	Expensive Unable to change plan intraoperatively Requires larger exposure Must protect predictive holes Longer lead-time to surgery Not suitable for mandible-first sequencing
Splint-only	Cheaper Faster production time Can be performed in-house Multiple intermediate position plans possible Allows some intraoperative changes to plan (especially vertical)	Reliant on accurate condylar seating Requires sufficient teeth present Introduction of operator error

## Data Availability

The original contributions presented in this study are included in the article. Further inquiries can be directed to the corresponding author.
